# Autophagy inhibition induced by EM-2 augments apoptosis via ROS-mediated ATM-Chk2-p53-p21 and MAPK pathway in lung and breast carcinoma

**DOI:** 10.3389/fphar.2025.1580217

**Published:** 2025-06-13

**Authors:** Jinxia Cheng, Ying Chen, Qi Chen, Ning Cao, Chuyu He, Yanjun Xu, Xiaoying Zhang, Qiang Li, Hong Hong, Jianwei Jiang, Yuechun Wang

**Affiliations:** ^1^ School of Medicine, Shenzhen Campus of Sun Yat-sen University, Sun Yat-sen University, Shenzhen, China; ^2^ Department of Physiology, Basic Medical College, Jinan University, Guangzhou, China; ^3^ Department of Biochemistry, Basic Medical College, Jinan University, Guangzhou, China; ^4^ Department of Radiation Oncology, Sun Yat-sen University Cancer Center, Guangzhou, China; ^5^ Department of Emergency, Sun Yat-Sen Memorial Hospital, Guangzhou, China; ^6^ Departments of Pathology, The Affiliated Panyu Central Hospital of Guangzhou Medical University, Guangzhou, China; ^7^ Department of General Surgery, The First Affiliated Hospital of Jinan University, Guangzhou, Guangdong, China; ^8^ Department of Breast Surgery, The Sixth Affiliated Hospital of Jinan University, Dongguan Eastern Central Hospital, Dongguan, China

**Keywords:** EM-2, autophagy, ER stress, cell cycle, apoptosis, lung cancer, breast cancer

## Abstract

Lung cancer (LC) and breast cancer (BC) are two common malignant tumors with the highest incidence rate in men and women worldwide, respectively. As the treatment effect of currently available therapies for LC and BC is unsatisfying, searching for new therapeutic drugs has become an urgent need to be addressed. EM-2, a natural sesquiterpene lactone isolated from *Elephantopus mollis* H.B.K., has been previously documented to exert anti-tumor effects on liver cancer by us. However, the underlying molecular mechanisms of its resistance to LC and BC have not been clearly elucidated. Thus, in the present study, we further investigated the anticancer effect of EM-2 on LC and BC with focusing on the involved molecular mechanisms. Our results suggest that EM-2 induces the impaired autophagy, which subsequently promotes ER stress-mediated apoptosis as well as ROS generation. ROS accumulation induced by EM-2 further simultaneously induces G2/M cell cycle arrest through ATM-Chk2-p53-p21 pathway and augments cell apoptosis via MAPK-mediated signaling pathway in LC and BC cells. These results may provide the experimental basis for future clinical application of EM-2 in the treatment for LC and BC.

## 1 Introduction

Around the global lung cancer, which has led to 125,070 deaths in America in 2024, is the primary cause of cancer death in men, and the second highest cause of cancer mortality in women, ranking behind only breast cancer (Leiter et al., 2023; Siegel et al., 2024). The current standard treatments for LC patients mainly consist of conventional radiotherapy and chemotherapy (Osmani et al., 2018), as well as emerging immunotherapy (Brahmer et al., 2018) and target therapy (Forde and Ettinger, 2013; Riely et al., 2024). However, most LC patients have poor prognosis due to the acquired drug resistance (Jackman et al., 2010), and experience treatment-related adverse events resulting from substantial drug toxicities (Schoenfeld et al., 2019; Oxnard et al., 2020), which remains a clinical hurdle for the treatment of LC. Breast cancer (BC) is the first cause of cancer-related death among women worldwide, with an estimate of approximately two million cases occurring each year (Bray et al., 2018). The limited therapeutic options, treatment resistance and disease recurrence are the three main causes of the curative failures for the treatment of BC (Cruceriu et al., 2020).

Given that the lack of effective targeted therapeutics for LC and BC patients results in their high recurrence rate, the development of more effective anti-cancer drugs with less side effects for the treatment of LC and BC is urgently needed. With the development of traditional Chinese medicine, some natural monomer products, being extracted from herbs, have been demonstrated its effectiveness in treating tumors (Ke et al., 2016). *Elephantopus mollis* H.B.K, an Elephantopus plant belonging to the composite family and named Baihuadidancao, is the most commonly used medicine in the south of the Yangtze River in China, and has been reported as an effective agent in the treatment of tumor, iron-induced damage, dysentery, etc. (Gibert-Tisseuil, 1998; Tabopda et al., 2008). We had identified 17 monomers from 10 kg of *Elephantopus mollis* H.B.K. Interestingly, we found that one of the monomers, EM-2, had prominent anti-proliferation effects on cancer cells. In our previous studies, we reported that EM-2 induces apoptosis by inhibiting autophagy and promoting G2/M phase arrest in hepatocellular carcinoma cells (Yang et al., 2021). We further reported that EM-2 can enhance the antitumor activity of epirubicin on breast cancer cells by blocking protective autophagy (Li et al., 2023). However, the molecular mechanism of anticancer effect of EM-2 on LC and BC remains obscure. Therefore, in the present study, we aimed to investigate the effect of EM-2 on autophagy, cell cycle and apoptosis in LC and BC cells, as well as elucidate the molecular mechanism involved.

## 2 Materials and methods

### 2.1 General experimental procedures

Optical rotations were measured by using a JASCO P-1020 polarimeter. UV spectra were recorded on a JASCO V-550 UV/VIS spectrophotometer. A JASCO FT/IR-480 plus FT-IR spectrometer was used for scanning the IR spectra with KBr pellets. HRESIMS data were determined using an Agilent 6210 ESI/TOF mass spectrometer. NMR spectra were obtained on a Bruker AV-400 spectrometer with TMS as internal standard. High performance liquid chromatography (HPLC) separations were performed using a COSMOSIL C18 preparative column (5 μM, 20 × 250 mm). Silica gel (200–300 mesh, Qingdao Marine Chemical Plant, Qingdao, P. R. China), ODS silica gel (50 μM, YMC, Kyoto, Japan), and Sephadex LH-20 (Pharmacia Biotech, Uppsala, Sweden) were used for column chromatography experiments. Silica gel GF254 plates (Yantai Chemical Industry Research Institute, Yantai, China) were used for thin-layer chromatography (TLC). All chemical reagents were purchased from Tianjin Damao Chemical Company (Tianjin, P. R. China).

### 2.2 Materials

The air-dried herbs of *E. mollis* were collected in Guangdong Province of China, in April 2013. The plant was authenticated by Zhenqiu Mai, the senior engineer of Guangdong Province. A voucher specimen (20130410) was storaged in the Institute of Traditional Chinese Medicine & Natural Products, Jinan University, Guangzhou, China.

### 2.3 Extraction and isolation

**FIGURE 1:**
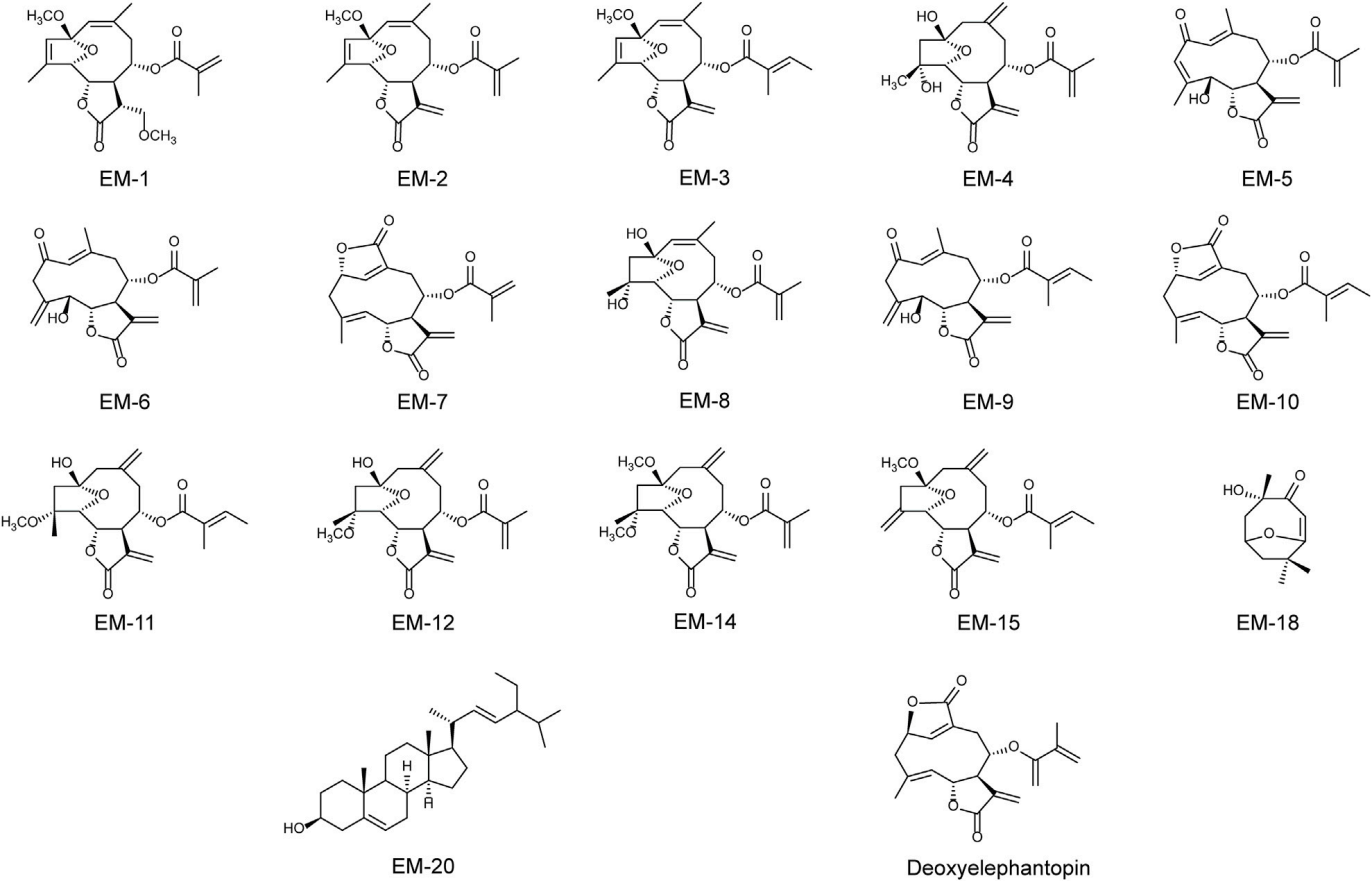
The chemical structures of EM-1, EM-2, EM-3, EM-4, EM-5, EM-6, EM-7, EM-8, EM-9, EM-10, EM-11, EM-12, EM-14, EM-15, EM-18, EM-20 and deoxyelephantopin.

The air-dried *E. mollis* (10 kg) were pulverized and extracted three times with 95% ethanol (30 L × 3) at room temperature. The residue (300 g) concentrated from the ethanol extract was further suspended in water (4 L) and then extracted with petroleum ether (4 L × 3) and ethyl acetate (4 L × 3). The petroleum ether extract (144 g) was subjected to silica gel column chromatography using a petroleum ether/ethyl acetate system (100:0→0:100, v/v) to yield seven fractions (Fr. A-G). Fr. B was further eluted by a silica gel column chromatography with a petroleum ether/ethyl acetate gradient (10:1→5:1, v/v) and then purified by Sephadex LH-20 to yield compounds EM-1 (6.8 mg) and EM-2 (235 mg). Fr. E was applied to a reversed silica gel column chromatography eluting with MeOH/H2O (40:60→100:0, v/v) and then separated by the preparative HPLC to yield compounds EM-3 (160 mg), EM-4 (5.6 mg), EM-5 (7.8 mg), EM-6 (12.3 mg), EM-7 (10.5 mg), EM-8 (6.5 mg), EM-9 (5.7 mg), EM-10 (16.8 g), EM-11 (6.5 mg), EM-12 (20.3 mg), EM-14 (6.9 mg), EM-15 (6.8 mg), EM-18 (6.7 mg), EM-20 (51 mg), and deoxyelephantopin (C19H20O6) (39.7 mg). Their chemical constructions were showed in Figure 1.

The following data were obtained on EM-2: white powder; [α] +33.5 (c 1.0, CH3OH), HRESIMS m/z 383.1465 [M + Na]+ (calcd for C20H24O6Na, 383.1465); UV (CH3OH) λmax 208 nm; IR (KBr) νmax 2,958, 2,924, 1,770, 1,716, 1,655, 1,431, 1,276, 1,157, 1,090 and 1,056 cm^−1^; 1H NMR (400 MHz, CDCl3) δH: 5.44 (1H, s, H-1), 5.66 (1H, s, H-3), 5.19 (1H, d, J = 4.0 Hz, H-5), 4.62 (1H, dd, J = 5.9, 4.0 Hz, H-6), 3.16 (1H, m, H-7), 5.20 (1H, s, H-8), 2.18 (1H, dd, J = 14.4, 4.4 Hz, H-9α), 3.68 (1H, d, J = 14.4 Hz, H-9β), 6.27 (1H, d, J = 2.6 Hz, H-13α), 5.75 (1H, d, J = 2.6 Hz, H-13β), 1.74 (3H, s, H-14), 1.69 (3H, s, H-15), 1.94 (3H, s, H-18), 5.66 (1H, s, H-19α), 6.12 (1H, s, H-19β), and 3.17 (2-OCH3); 13C NMR (100 MHz, CDCl3) δC: 127.3 (C-1), 114.2 (C-2), 129.2 (C-3), 139.2 (C-4), 85.9 (C-5), 79.4 (C-6), 39.1 (C-7), 76.7 (C-8), 32.6 (C-9), 133.9 (C-10), 135.2 (C-11), 169.1 (C-12), 127.2 (C-13), 28.6 (C-14), 13.1 (C-15), 165.8 (C-16), 136.3 (C-17), 18.4 (C-18), 126.6 (C-19), and 49.7 (2-OCH3).

### 2.4 Chemicals and reagents

The monomer EM-2 was obtained from the Institute of Traditional Chinese Medicine and Natural Products of Jinan University. EM-2 was dissolved in dimethyl sulfoxide (DMSO) (Sigma-Aldrich, CA) at the concentration of 100 mM. Annexin V- FITC Apoptosis Detection Kit, Cell Cycle Detection Kit, JC-1 Apoptosis Detection Kit, MTT Cell Proliferation Detection Kit, EdU Cell Proliferation Detection Kit, Trypsin-EDTA and Cytotoxicity Detection Kit were purchased from KeyGEN BioTECH (Jiangsu, China). Reactive Oxygen Species Assay Kit were purchased from Beyotime (Shanghai, China). Z-VAD-FMK, SB203580, SP600125, Bafilomycin A1 (Baf-A1) and N-acetyl-l-cysteine (NAC) were obtained from Selleck (Houston, United States). Immobilon electrochemilu-minescence (ECL) Kit and polyvinylidene fluoride (PVDF) were obtained from Millipore (MA, United States). Antibodies against Caspase-9, CL-caspase-9, PARP, CL-PARP, Caspase-8, CL-caspase-8, Mcl-1, Bid, Bax, Bak, Bim, PUMA, ATM, p-ATM, Chk2, p-Chk2, p53, p-p53 (S15), p21, Cdc2, p-Cdc2 (Tyr-15), CyclinD1, CyclinB1, CyclinA2, Myt1, p-Wee1, ERK, p-ERK, JNK, p-JNK, p38, p-p38, CHOP, BiP, PDI, p62, Beclin-1, and LC3 were purchased from Cell Signaling Technology (Boston, MA). Antibodies against cathepsin D were purchased from Santa Cruz Biotechnology (Boston). The other chemicals were obtained from Advansta (CA, United States) and Sigma (Saint Louis, MO, United States).

### 2.5 Cell culture

The human lung carcinoma cell line A549, breast carcinoma cell line MCF-7, MDA-MB-231, MDA-MB-468, human hepatoma cell line HepG2, normal human lung epithelial cells (BEAS-2B) and human skin fibroblasts (HSF) were obtained from the American Type Culture Collection (Rockville, MD) and were cultivated in DMEM supplemented with 10% fetal bovine serum in a 5% CO_2_ humidified atmosphere at 37°C. Fetal bovine serum, DMEM, trypsin and EDTA were purchased from Gibco.

### 2.6 MTT assay

A549 cells (7.5 × 10^4^ cells/mL) and MCF-7 cells (5 × 10^4^ cells/mL) were seeded in 96-well plates overnight and then treated with EM-2. After 48 h, 20 μL MTT was added to each well for 4 h at 37°C. Absorbance was measured at 570 nm using a microplate reader (Bio-Rad Laboratories, Hercules, CA). The experiment was independently repeated three times as biological replicates.

### 2.7 Colony formation assay

A549 and MCF-7 cells in the logarithmic growth phase were seeded in a six-well plate at a density of 800 cells per well at 37°C in a 5% CO_2_ incubator overnight. Then, A549 and MCF-7 cells were treated with EM-2 for 48 h. After removing the supernatant, A549 and MCF-7 cells were cultured in drug-free medium for 7 and 14 days, respectively. After discarding the supernatants, cells were washed three times with phosphate-buffered saline (PBS) and fixed with 4% paraformaldehyde for 10 min. Finally, the cells were stained in 0.5% crystal violet for 30 min. Images of the colonies were captured by a digital camera. The experiment was independently repeated three times as biological replicates.

### 2.8 5-Ethynyl-2′-deoxyuridine incorporation analysis

A549 cells (7.5 × 10^4^ cells/mL) and MCF-7 cells (5 × 10^4^ cells/mL) were seeded in 96-well plates. After adherence and EM-2 treatment for 24 h, the DNA synthesis rate in A549 and MCF-7 cells was determined by five-ethynyl-2′-deoxyuridine (EdU) incorporation analysis using an EdU cell proliferation detection kit according to the manufacturer’s instructions. The experiment was independently repeated three times as biological replicates.

### 2.9 Analysis of apoptosis by annexin V-FITC/PI staining

A549 and MCF-7 cells were seeded in six-well plates at a density of 2.0 × 10^5^ cells per well. After adherence and EM-2 treatment for 48 h, cells were harvested and washed with phosphate-buffered saline (PBS) twice. Then, 5 μL Annexin V-FITC and 5 μL propidium iodide (PI) were added to the cells, and the cells were incubated for another 15 min according to the protocol of the Annexin V-FITC/PI Kit. After treatment, the cells were analyzed using a flow cytometer (Becton, Dickinson and Company, VT). The experiment was independently repeated three times as biological replicates.

### 2.10 Cell cycle distribution analysis

A549 and MCF-7 cells were seeded in six-well plates at a density of 2.0 × 10^5^ cells per well and allowed to attach for 24 h. After EM-2 treatment for 48 h, the cells were harvested, washed three times with cold PBS, and fixed in 70% ethanol overnight at 4°C. Cells were then washed twice with cold PBS and incubated for 30 min in the dark with PI solution (working concentrations: 50 μg/mL PI and 100 μg/mL RNase A). Next, cells were analyzed on a flow cytometer (Becton, Dickinson and Company, VT). Approximately 10,000 cells were analyzed per sample.

### 2.11 Mitochondrial membrane potential (DΨm) assay by JC-1 staining

A549 and MCF-7 cells at logarithmic growth were sowed into 6-well plates at a density of 2.0 × 10^5^ cells/well and incubated at 37°C overnight; After EM-2 treatment for 24 h, cells were washed by PBS, and centrifuged (1,000 rpm/min, 5 min). After discarding the supernatant, cells were incubated in 200 μL of JC-1 dye stuff for 20 min at 37°C, followed by washing with 1× Incubation Buffer twice and detected by a FACSCanto flow cytometer (BD, United States). The experiment was independently repeated three times as biological replicates.

### 2.12 Detection of intracellular ROS concentration

A549 and MCF-7 cells in the logarithmic growth phase were seeded in 6-well plates at a density of 2.0 × 10^5^ cells/well and treated with increasing concentrations of EM-2 for 24 h. The cells were then incubated with 200 μL of 10 μM DCFH-DA for 20 min at 37°C in the dark. DCFH-DA, a non-fluorescent cell-permeable probe, is hydrolyzed by intracellular esterases to DCFH (non-fluorescent and membrane-impermeable), which is subsequently oxidized by reactive oxygen species (ROS) to fluorescent DCF. After washing the cells twice with 1× PBS, ROS levels were quantified based on DCF fluorescence intensity using a FACSCanto flow cytometer (BD, United States). The experiment was independently repeated three times as biological replicates.

### 2.13 Transcriptome analysis

A549 cells at logarithmic growth were sowed into 6-well plates at a density of 5.0 × 10^6^ cells per well and incubated at 37°C overnight. After EM-2 treatment for 24 h, the cells were washed by PBS, and centrifuged (1,000 rpm/min, 5 min). Total RNA was isolated from A549 cells, using TRIzol Reagent (Invitrogen Corporation) according to the manufacturer’s protocol. RNA libraries for RNA-Seq were prepared using MGIEasy RNA library preparation kit following manufacturer’s protocols. Then samples were sequenced by The Beijing Genomics Institute (BGI) (Shenzhen, China), using the BGISEQ-500 system. The heatmap was generated by heatmap (v1.0.8) according to the gene expression in different samples. GO (http://www.geneontology.org/) and KEGG (https://www.kegg.jp/) enrichment analysis of annotated different expressed gene was performed by Phyper (https://en.wikipedia.org/wiki/Hypergeometric_distribution) based on Hypergeometric test. The significant levels of terms and pathways were corrected by Q value with a rigorous threshold (Q value ≤0.05) by Bonferroni.

### 2.14 Western blot analysis

The cells were seeded in six-well plates at a density of 2.0 × 10^5^ cells per well; after attachment, A549 cells were treated with different concentrations of EM-2 (0.04% DMSO, 3, 6, and 12 μM) while MCF-7 cells were treated with increasing dose of EM-2 (0.04% DMSO, 2, 4, and 8 μM) for 24 h. After the cells were collected, they were homogenized in 1 × RIPA buffer (Cell Signaling Technology, MA, United States), and protein concentrations were quantified using a BCA protein assay kit (Sangon Biotch, China) according to the manufacturer’s instructions. Equal amounts of protein (30 μg) were separated by SDS-PAGE (10%–15%) and transferred to a PVDF membrane. The membranes were blocked in 5% nonfat milk for 1 h at room temperature and incubated overnight at 4°C with specific primary antibodies, followed by incubation with a secondary antibody for 1 h at room temperature. After washing for three times, the membrane was covered with Immobilon ECL luminescent liquid and visualized using the Gel Image System (UVItec Ltd., Cambridge, United Kingdom).

### 2.15 Statistical analysis

Measurement data were expressed as the mean ± SD and evaluated using GraphPad Prism (Version 5.0 GraphPad software, San Diego, California, United States). Data were analyzed using SPSS (Version 15.0.1, Palo Alto, California) with the Independent-Samples *t*-Test, one-way ANOVA, and Student–Newman–Keuls (SNK) test. P < 0.05 was considered statistically significant.

## 3 Results

### 3.1 EM-2 was the most potent anti-cancer compound among the *Elephantopus mollis* H.B.K. monomers

**FIGURE 2:**
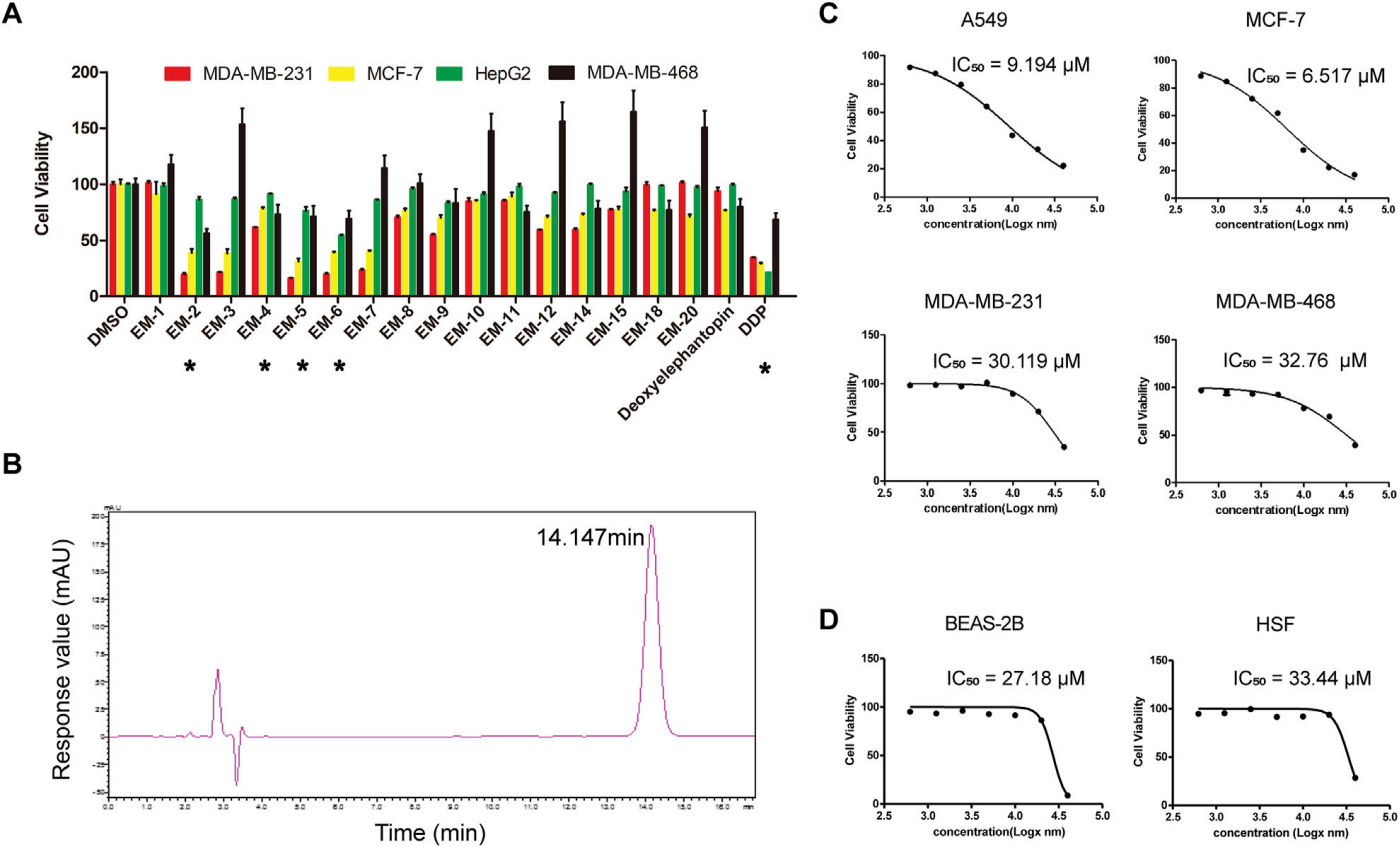
EM-2 was the most potent anti-cancer compound among the *Elephantopus mollis* H.B.K. monomers. **(A)** MDA-MB-231, MDA-MB-468, MCF-7 and HepG2 cells were respectively treated with each compound (10 μM) for 48 h and MTT assay was performed. DMSO and DDP were used as the negative and positive controls, respectively. Asterisks indicate statistically significant differences to the control group; *p < 0.05. **(B)** Compound EM-2 was analyzed by HPLC (CH_3_OH:H_2_O:CH_3_COOH = 75:25:0.01, 1450.23 psi, 25.12°C, 210 nm). The purity of the compound EM-2 was over 95%. **(C)** IC50 values of EM-2 for A549, MCF-7, MDA-MB-231 and MDA-MB-468 cells *in vitro*. Cells were treated with various concentrations of EM-2 (0.625, 1.25, 2.5, 5, 10, 20, or 40 μM), and DMSO (0.04%) was used as the vehicle control. **(D)** IC50 values of EM-2 for BEAS-2B and HSF cells *in vitro*. Cells were treated with various concentrations of EM-2 (0.625, 1.25, 2.5, 5, 10, 20, or 40 μM), and DMSO (0.04%) was used as the vehicle control.

To evaluate the effects of the 17 compounds obtained from *E. mollis* H.B.K. on cancer cell proliferation, a hepatocarcinoma cell line, HepG2, and three BC cell lines, including MDA-MB-231, MDA-MB-468 and MCF-7, were respectively treated with each compound (10 μM) for 48 h, and then a MTT assay was performed. As indicated in Figure 2A and Table 1, EM-2, EM-4, EM-5 and EM-6 were proved to have growth inhibitory effect on cancer cells, with EM-2 showing the most potent cytotoxicity on cancer cells and the highest abundance among the four compounds, prompting us to choose EM-2 as the study object. Moreover, EM-2 had similar effects as the chemotherapeutic drug, cisplatin (DDP), on these cancer cells (Figure 2A). Additionally, HPLC analysis of EM-2 only had one major peak, indicating that this compound was homogeneous and its purity was over 95% (Figure 2B).

**TABLE 1:** Monomers content extracted from 10 kg *Elephantopus mollis*.

Monomer	Content (mg)
EM-2	235
EM-4	5.6
EM-5	7.8
EM-6	12.3

### 3.2 EM-2 inhibited cell proliferation in A549 and MCF-7 cells

**FIGURE 3:**
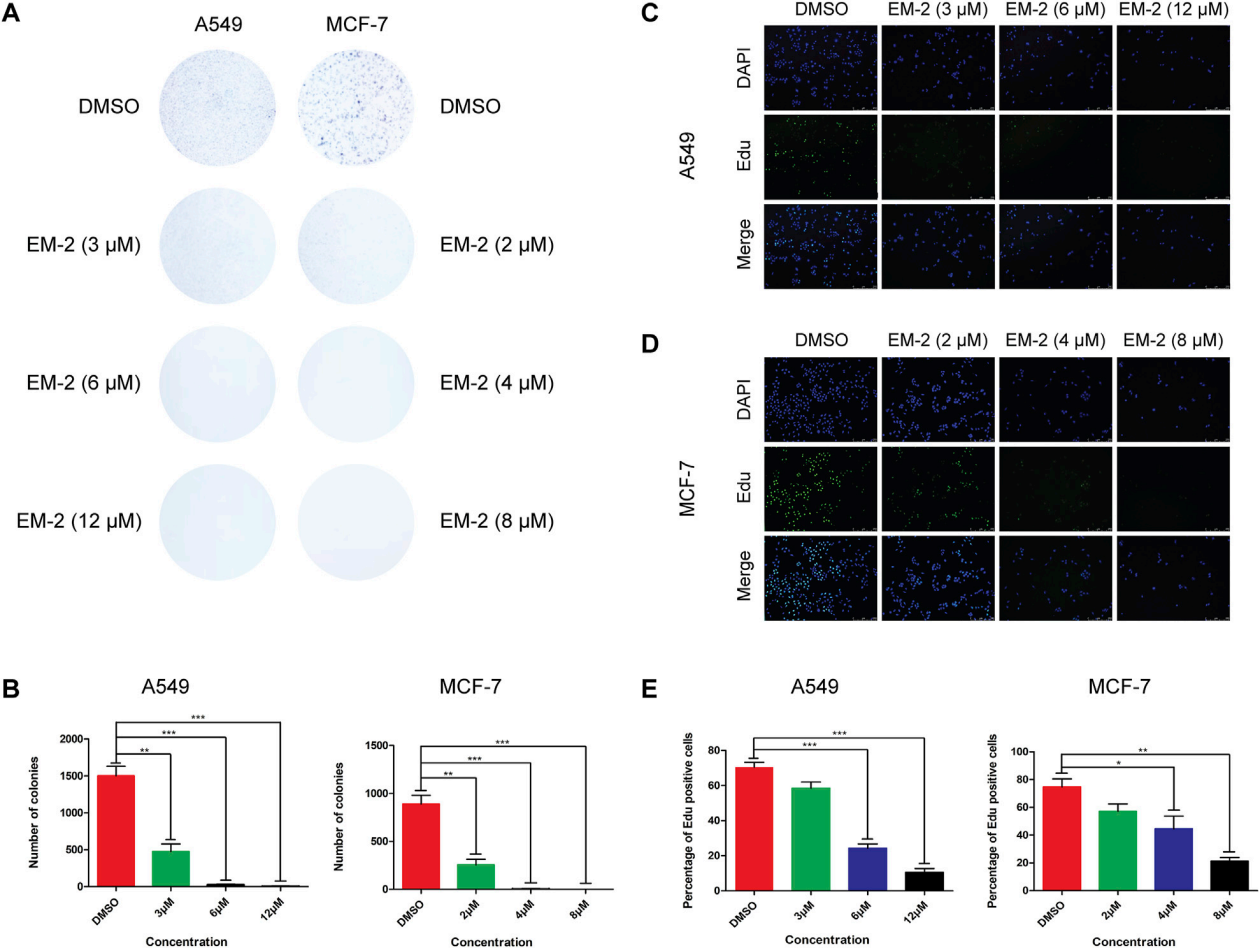
Growth-inhibitory effect of EM-2 on A549 and MCF-7 cells. **(A,B)** A549 and MCF-7 cells were exposed to various concentrations of EM-2 (3, 6, 12 μM in A549 cells, and 2, 4, 8 μM in MCF-7 cells) for 48 h. After removing the supernatant, A549 and MCF-7 cells were cultured in drug-free medium for 7 and 14 days, respectively. DMSO (0.04%) was used as the vehicle control. Cell survival was evaluated by colony formation assays. **(C,D)** Cell proliferation was determined by EdU staining, and the green dots represent the population of newborn cells. A549 and MCF-7 cells were detected under confocal microscopy after being exposed to EM-2 (3, 6, 12 μM in A549 cells, and 2, 4, 8 μM in MCF-7 cells) for 24 h. DMSO (0.04%) was used as the vehicle control. Scale bar = 250 μM. **(E)** Percentage of Edu positive cells. Asterisks indicate statistically significant differences from each other; *p < 0.05, **p < 0.01, ***p < 0.001.

To determine the half maximal inhibitory concentration (IC50) of EM-2, LC cells, A549, and BC cells, MDA-MB-231, MDA-MB-468 and MCF-7, were treated with increasing concentrations of EM-2 (0–40 μM) for 48 h, and then MTT assay was performed. As indicated in Figure 2C, the IC50 values of A549, MCF-7, MDA-MB-231, MDA-MB-468 were 9.194, 6.517, 30.119 and 32.760 μM, respectively, suggesting that EM-2 had significant inhibitory effect on A549 and MCF-7 cell proliferation. To further confirm the inhibitory effect of EM-2 on A549 and MCF-7 cell proliferation, we conducted colony formation and EdU assay. As expected, EM-2 dramatically inhibited colony formation (Figures 3A,B) and significantly decreased DNA synthesis (Figures 3C–E) in LC and BC cells in a dose-dependent manner. To evaluate the potential cytotoxicity of EM-2 on normal cells, we conducted toxicity assays using normal human lung epithelial cells (BEAS-2B) and human skin fibroblasts (HSF). The IC50 values of BEAS-2B and HSF were 27.18 μM and 33.44 μM, demonstrating significantly lower toxicity toward normal cells (Figure 2D). Taken together, these results demonstrated that EM-2 could significantly inhibit the proliferation of A549 and MCF-7 cells.

### 3.3 EM-2 triggered caspase-dependent apoptosis in A549 and MCF-7 cells

**FIGURE 4:**
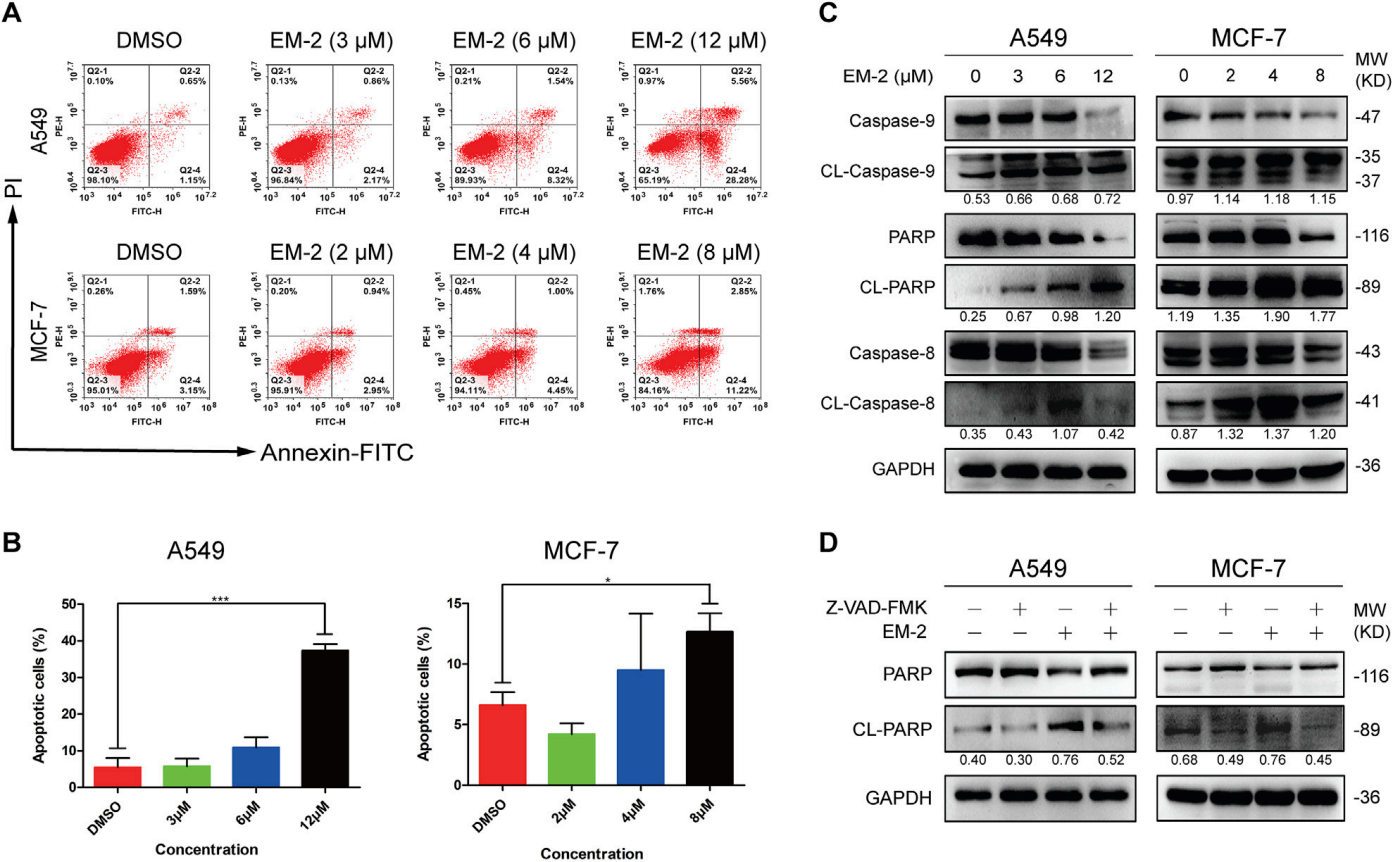
EM-2 induced apoptosis in A549 and MCF-7 cells. **(A)** A549 cells were treated with different concentrations of EM-2 (3, 6, 12 μM) while MCF-7 cells were treated with increasing dose of EM-2 (2, 4, 8 μM) for 48 h. DMSO (0.04%) was used as the vehicle control. Then, cells were analyzed using PI/Annexin V-FITC flow cytometry. **(B)** The percentage of apoptotic cells are presented as the mean ± SD of triplicate determinations (vs. control, *P < 0.05, ***P < 0.001). **(C)** A549 and MCF-7 cells were treated with different concentrations of EM-2 for 24 h; expression of apoptosis-related proteins was analyzed by Western blotting. **(D)** Cells were pretreated with 20 μM Z-VAD-FMK for 6 h, exposed to 8 μM (MCF-7 cells) or 12 μM (A549 cells) EM-2, followed by incubation for another 24 h. Then the expression of apoptosis-related proteins were detected by Western blotting.

To investigate whether the inhibitory effect of EM-2 on LC and BC cell proliferations was caused by cell apoptosis, Annexin V-FITC/PI double staining assay was performed. As shown in Figures 4A,B, EM-2 treatment increased the percentage of apoptotic A549 and MCF-7 cells in a dose-dependent manner. Also, Western blot analysis clearly showed that the protein levels of Caspase-9, PARP and Caspase-8 were downregulated, while CL-caspase-9, CL- PARP and CL-caspase-8 were upregulated after EM-2 treatment, which further confirmed the occurrence of cell apoptosis induced by EM-2 in both LC and BC cells (Figure 4C). Moreover, pretreatment with Z-VAD-FMK, an inhibitor of the caspase family, significantly attenuated EM-2 induced cell apoptosis (Figure 4D). Our results demonstrated that EM-2 promoted apoptosis through the caspase dependent pathway in A549 and MCF-7 cells.

### 3.4 EM-2 promotes apoptosis via the mitochondrial signaling pathway in A549 and MCF-7 cells

**FIGURE 5:**
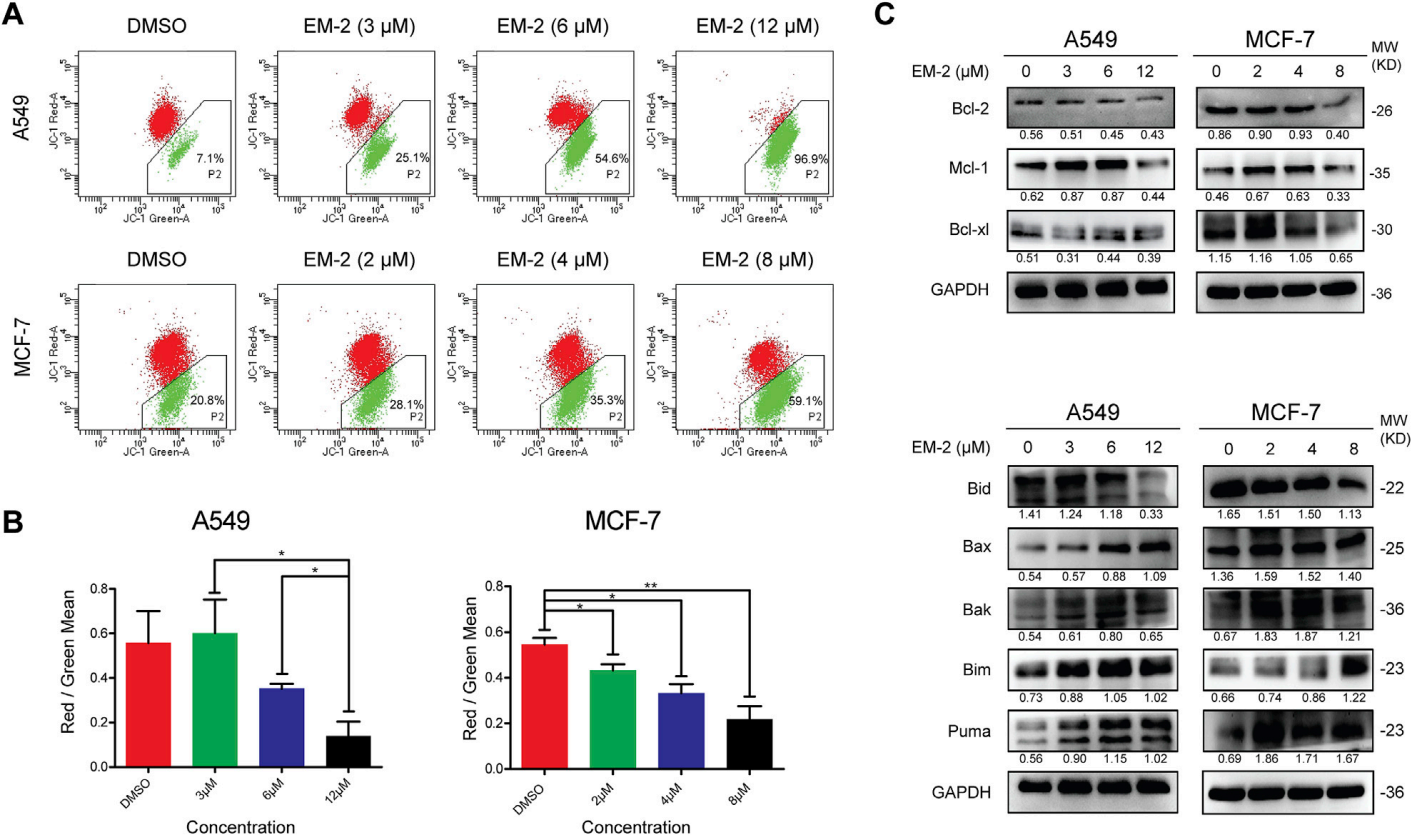
EM-2 induced apoptosis through the mitochondrial signaling pathway in A549 and MCF-7 cells. **(A)** Following EM-2 treatment at a series of concentrations (3, 6, 12 μM) in A549 cells and that (2, 4, 8 μM) in MCF-7 cells for 24 h, mitochondrial membrane potential was detected by JC-1 assay using flow cytometry. DMSO (0.04%) was used as the vehicle control. **(B)** The Red/Green Mean are presented as the mean ± SD of triplicate determinations (vs. control, *P < 0.05, **P < 0.01). **(C)** A549 and MCF-7 cells were treated with different concentrations of EM-2 for 24 h; the expression of Bcl-2-family proteins was analyzed by Western blotting.

Previous studies have reported that mitochondrial apoptosis, a process of programmed death that kills cancer using the enemy within, is the most commonly deregulated form of cell death in cancer (Xia et al., 2019). To determine the role of the mitochondrial endogenous pathway in the promotion of apoptosis, the mitochondrial transmembrane potential was measured using JC-1 staining. The P2 percentage of A549 cells increased from 7.1% to 96.9% and that of MCF-7 cells increased from 20.8% to 59.1% (Figures 5A,B), suggesting that the mitochondrial membrane potential decreased significantly after EM-2 treatment in A549 and MCF-7 cells. Furthermore, an obvious increase in the expression of Bax, Bak, Bim and PUMA and decrease in the expression of Bcl-2, Mcl-1 and Bid were found upon treatment with different concentration of EM-2, while the level of Bcl-xl decreased in MCF-7 cells but had no change in A549 cells (Figure 5C). Taken together, these results suggested that EM-2 induced apoptosis of A549 and MCF-7 cells via the mitochondrial signaling pathway.

### 3.5 EM-2 induced incomplete autophagy by inhibiting the acidification of autolysosomes in A549 and MCF-7 cells

**FIGURE 6:**
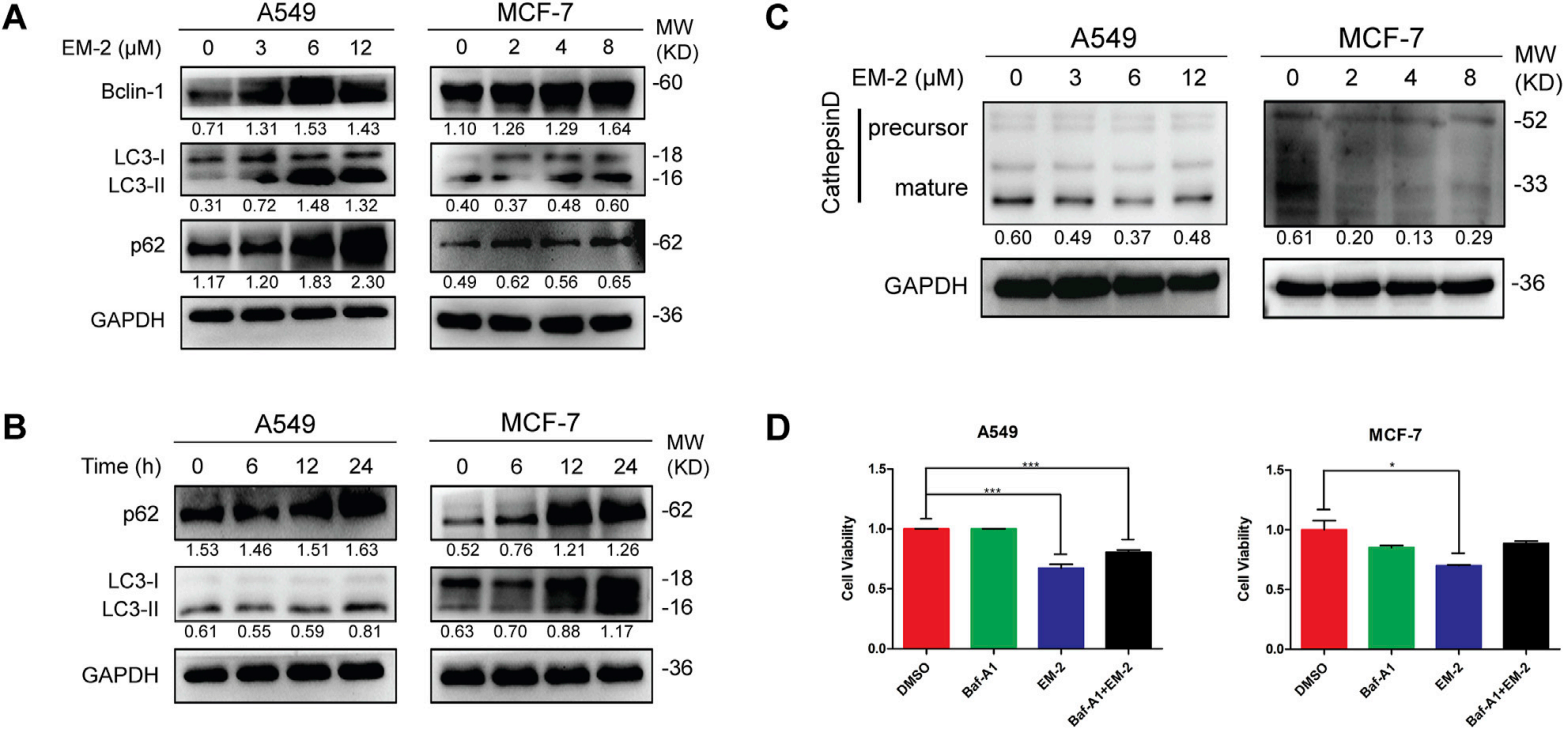
EM-2 induced the impaired autophagy in A549 and MCF-7 cells. **(A,C)** In cells exposed to four different concentration of EM-2, Western blotting was performed to evaluate the protein expression. **(B)** A549 and MCF-7 cells were treated with 6 μM and 4 μM EM-2 for different times, respectively; the expression level of p62 and LC3-I/II proteins was analyzed by Western blotting. **(D)** Cells were pretreated with 100 nM Baf-A1 for 6 h, followed by exposure to 8 μM (MCF-7 cells) or 12 μM (A549 cells) EM-2 and 24 h incubation. Then cell viability was detected by MTT assay. Asterisks indicate statistically significant differences from each other; *p < 0.05, ***p < 0.001.

Next, we determined whether autophagy played a crucial role in the EM-2-induced inhibition of A549 and MCF-7 cell proliferation. LC3 and Beclin-1 are two of the known markers for autophagy initiation. The accumulation of LC3-II in a dose- and time-dependent manner and the increase of Beclin-1 in a dose-dependent manner upon EM-2 treatment suggested the induction of autophagy (Figures 6A,B). p62, an autophagic substrate, can be degraded when lysosome is acidified during the maturation of autolysosomes. The increase of p62 in a dose- and time-dependent manner suggested that EM-2 induced an incomplete autophagy in A549 and MCF-7 cells (Figures 6A,B).

Acidification of autolysosomes plays a crucial role in activating cathepsin and affecting proteolysis of substrates, which contributes to the maintenance of autophagic flux. Procathepsin D, a short-lived inactive precursor of lysosomal aspartyl protease, is cleaved to yield mature cathepsin D (CTSD), mainly depending on acidification in autolysosomes. Interestingly, expression of the mature CTSD was downregulated while the precursor of CTSD remained unchanged after EM-2 treatment in A549 and MCF-7 cells (Figure 6C), suggesting that EM-2 suppressed autophagy via inhibiting the acidification of autolysosomes.

To examine the effect of autophagy inhibition on A549 and MCF-7 cells, cells were treated with bafilomycin A1 (Baf-A1), a selective inhibitor of the vacuolar-type H^+^ -translocating ATPase, and detected by MTT assay (Figure 6D). As we observed, Baf-A1 attenuated EM-2-induced cell death and promoted cell viability in A549 and MCF-7 cells. These data suggested that the impaired autophagy induced by EM-2 promoted cell viability in A549 and MCF-7 cells.

### 3.6 EM-2 activated ER stress-mediated intrinsic apoptosis in response to the impaired autophagy

**FIGURE 7:**
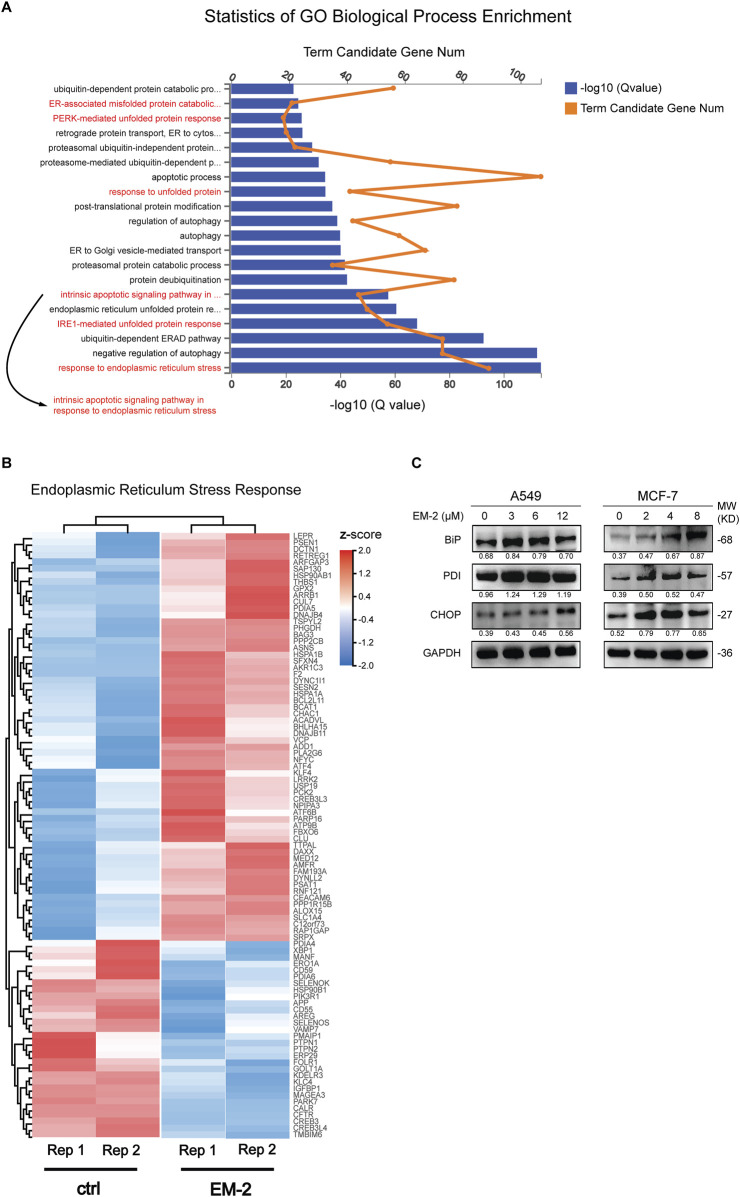
Autophagy inhibition induced by EM-2 was responsible for activation of ER stress. **(A)** RNA-seq was carried out to identify the transcriptional profile in A549 cells treated with 0.04% DMSO or 6 μM EM-2. Go Biological Process Enrichment analysis results showed the enriched biological processes activated by EM-2. **(B)** Heatmap depicted the mRNA levels of genes involved in ER stress response. Blue, decreased mRNA expression; red, elevated mRNA expression. **(C)** A549 and MCF-7 cells were treated with different concentrations of EM-2 for 24 h; the expression levels of ER stress related proteins was analyzed by Western blotting.

To further uncover the molecular mechanism of EM-2-inhibited autophagy, transcriptome profiling by RNA sequencing (RNA-Seq) was conducted. Result of the GO Biological Process (GO-BP) Enrichment analysis showed that the process of response to endoplasmic reticulum stress was remarkably significant among the biological processes activated by EM-2 (Figure 7A). Moreover, heatmap of the 92 genes involving in ER stress was constructed to compare the transcriptome between control and EM-2-treated A549 cells. As shown in Figure 7B, among these 92 genes, the expression of 62 genes were upregulated while the expression of the rest of them were downregulated following EM-2 treatment. Additionally, the biological process of response to unfolded protein was activated after EM-2 treatment (Figure 7A), which led to the induction of ER stress. The result from the GO-BP Enrichment analysis also suggested that PERK-mediated unfolded protein response and IRE1-mediated unfolded protein response were activated following EM-2 treatment (Figure 7A). Consistent with these results, the expression levels of BiP, PDI and CHOP were dramatically increased upon EM-2 in a dose-dependent manner (Figure 7C). Increasing evidence suggests that the impaired autophagy inhibits the removal of misfolded proteins and disrupts cell homeostasis, which eventually induces apoptosis via endoplasmic reticulum stress (Moriya et al., 2013). Similar to previous findings, GO-BP Enrichment analysis results showed that 678 genes were significantly enriched in the biological process of intrinsic apoptotic signaling pathway in response to endoplasmic reticulum stress, supporting that ER stress-related apoptosis induced by EM-2 was activated in response to the impaired autophagy.

### 3.7 EM-2 induced ROS production in response to the impaired autophagy

**FIGURE 8:**
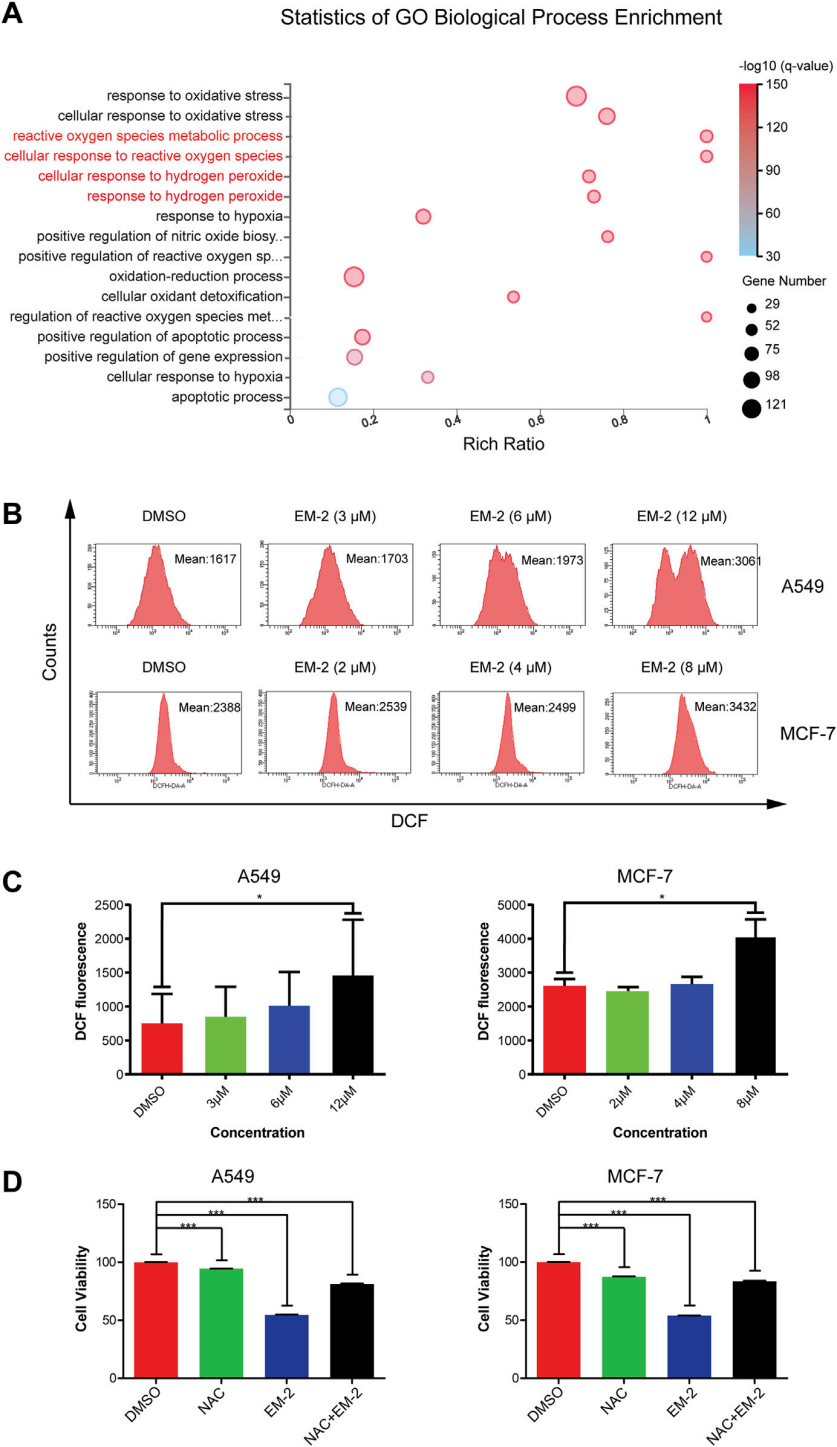
Autophagy inhibition induced by EM-2 was responsible for ROS production. **(A)** Go Biological Process Enrichment analysis of DEGs. Rich Ratio = Term Candidate Gene Num/Term Gene Num. Greater Rich Ratio means greater intensiveness. The size of the bubble represents the number of differentially expressed genes annotated to the GO Term. The color represents the enriched value of -log10 (Q-value). Red, lower Q-value; blue, higher Q-value. A lower Q-value represents greater intensity. **(B,C)** After EM-2 treatment (3, 6, 12 μM in A549 cells and 2, 4, 8 μM in MCF-7 cells) for 24 h, cells were stained with DCFH-DA and measured by flow cytometry. DMSO (0.04%) was used as the vehicle control. The data are expressed as the mean fluorescence intensity of three independent experiments. *P < 0.05 versus control. **(D)** A549 cells were treated with 1 mM NAC and 9 μM EM-2 for 48 h, while MCF-7 cells were treated with 1 mM NAC and 6 μM EM-2 for 48 h. MTT assay was performed to detect cell viability. The data were shown as the mean ± SD of three independent experiments. *** means p-value <0.001.

It has been reported that the impaired autophagy inhibits the removal of damaged and aging organelles, resulting in an increase in intracellular ROS level as well as mitochondrial ROS level (Guo et al., 2014). Consistent with the previous study, GO-BP Enrichment analysis results showed that 634 genes were markedly enriched in some biological processes, including cellular response to hydrogen peroxide, cellular response to reactive oxygen species, cellular response to oxidative stress, apoptotic process and some metabolic processes, supporting that EM-2 could stimulate ROS generation (Figure 8A). Cellular hydrogen peroxide response led to the accumulation of ROS, resulting in cellular oxidative stress response and induction of cell apoptosis. Moreover, our results showed that EM-2 treatment resulted in an increase in the intensity of DCF fluorescence, which was correlated with intracellular ROS (Figures 8B,C).

To further confirm that EM-2 induced ROS generation in A549 and MCF-7 cells, cells were treated with N-acetylcysteine (NAC), an antioxidant, and detected by MTT assay (Figure 8D). As we observed, NAC attenuated EM-2-induced cell death and promoted cell viability in A549 and MCF-7 cells (P < 0.001). These results suggested that EM-2 triggered significant ROS accumulation in response to defective autophagy in A549 and MCF-7 cells.

### 3.8 EM-2 induced G2/M phase arrest via regulating ROS-mediated ATM-Chk2-p53-p21 pathway in LC and BC cells

**FIGURE 9:**
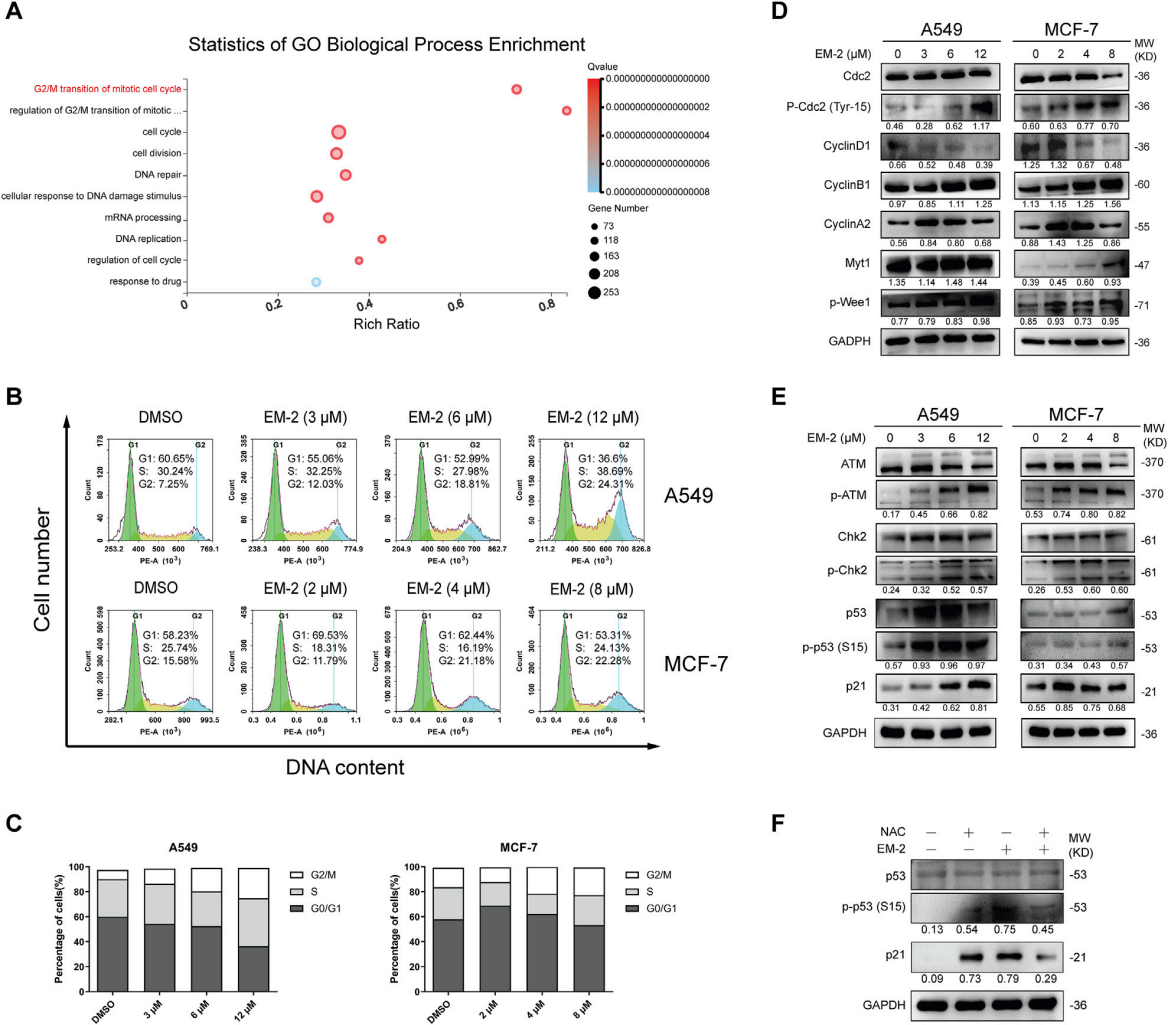
EM-2 induces G2/M arrest in response to ROS generation in LC and BC cells. **(A)** Go Biological Process Enrichment analysis shows 10 prominent biological processes affected by EM-2 treatment. Rich Ratio = Term Candidate Gene Num/Term Gene Num. Greater Rich Ratio means greater intensiveness. The size of the bubble represents the number of differentially expressed genes annotated to the GO Term. The color represents the enriched Q-value and a lower Q-value represents greater intensity. **(B,C)** A549 cells were treated with different concentrations of EM-2 (3, 6, 12 μM) while MCF-7 cells were treated with increasing dose of EM-2 (2, 4, 8 μM) for 48 h. DMSO (0.04%) was used as the vehicle control. DNA content was analyzed by flow cytometry using PI staining. The percentage of cells in each phase of cell cycle was calculated using GraphPad Prism software. **(D,E)** A549 and MCF-7 cells were incubated with different concentrations of EM-2 for 24 h; expression of cell cycle-associated proteins was analyzed by Western blotting. **(F)** A549 cells were pretreated with 5 mM NAC for 6 h, followed by exposure to 12 μM EM-2 and incubation for another 24 h, then proteins were detected by Western blotting.

Increasing evidence suggests that ROS generation may induce ATM-mediated DNA Damage and cause G2/M cell cycle arrest (Prasad Tharanga Jayasooriya et al., 2018). Thus, we further investigated whether EM-2-induced ROS production affected cell cycle distribution in A549 and MCF-7 cells. As expected, GO-BP Enrichment analysis result showed that the process of G2/M transition of mitotic cell cycle was notably significant among the biological processes activated by EM-2 (Figure 9A). Consistent with this result, we found that the fraction of cells in G2/M phase increased in A549 and MCF-7 cells (Figures 9B,C). Moreover, following EM-2 treatment, the expression of CyclinD1 was noticeably reduced, while the expression of p-Cdc2 (Tyr15), CyclinB1, CyclinA2, Myt-1, p-Wee1 were markedly increased. Meanwhile, the expression of Cdc2 was downregulated in MCF-7 cells and remained unchanged in A549 cells (Figure 9D). These results indicated that EM-2 induces G2/M phase arrest in A549 and MCF-7 cells. Next, we investigated whether G2/M cell cycle arrest was triggered by the accumulation of ROS via ATM-dependent DNA damage signaling pathway. At first, with increasing EM-2 concentrations, there was no obvious change in ATM and Chk2 expression; however, p-ATM, p-Chk2, p53, p-p53 (S15) and p21 levels were elevated, indicating that EM-2 blocked cell cycle progression of A549 and MCF-7 cells at G2/M phase via ATM-Chk2-p53-p21 signaling pathway (Figure 9E). To further confirm that ROS induction was the upstream regulator of EM-2-induced G2/M cell cycle arrest, an antioxidant, N-acetylcysteine (NAC), was used. As expected, EM-2-induced p-p53 (S15) and p21 activations were markedly inhibited by the treatment with NAC, while p53 remained unchanged in A549 cells (Figure 9F), suggesting that EM-2 induced G2/M phase arrest through ROS-mediated ATM-Chk2-p53-p21 pathway in A549 and MCF-7 cells.

### 3.9 EM-2 triggered apoptosis via regulating ROS-mediated MAPK pathway in LC and BC cells

**FIGURE 10:**
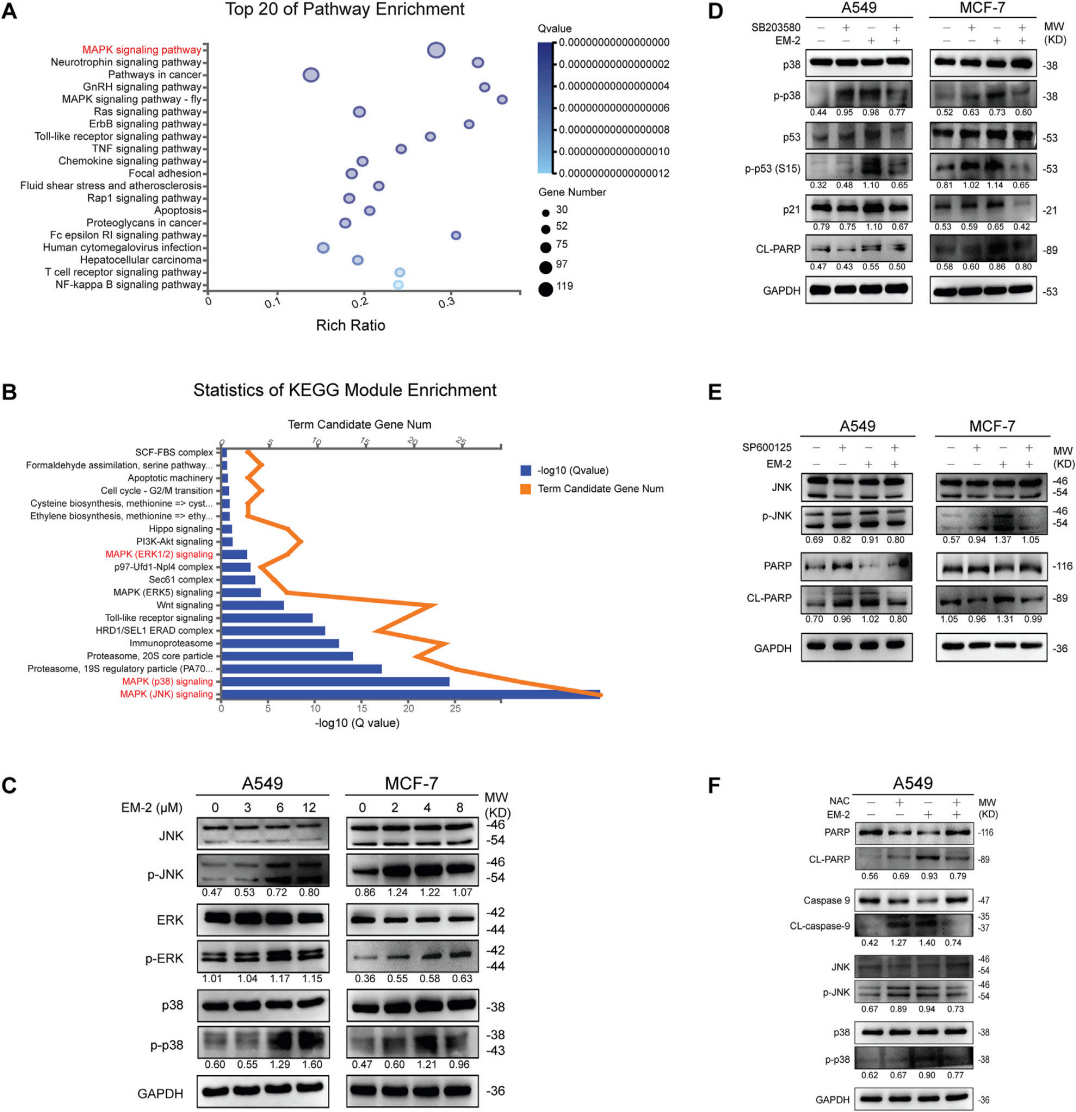
EM-2 induced MAPK-mediated apoptosis in response to ROS generation in LC and BC cells. **(A)** Kyoto Encyclopedia of Genes and Genomes (KEGG) pathway enrichment analysis shows the top 20 biological processes affected by EM-2 treatment based on the DEGs in A549 cells. In the scatter plot, Rich Ratio = Term Candidate Gene Num/Term Gene Num. Greater Rich Ratio means greater intensiveness. The size of the bubble represents the number of differentially expressed genes annotated to the KEGG pathway. The color represents the enriched Q-value, and a lower Q-value represents greater intensity. The MAPK signaling pathway was the most affected biofunction after EM-2 treatment. **(B)** KEGG Module Enrichment analysis of DEGs. MAPK (JNK) signaling, MAPK (p38) signaling and MAPK (ERK1/2) signaling were three of the most affected biofunctions after EM-2 treatment. **(C)** A549 and MCF-7 cells were treated with different concentration of EM-2 for 24 h; expression of MAPK-family proteins was analyzed by Western blotting. **(D)** Cells were pretreated with 4 μM SB203580 for 6 h, followed by exposure to 8 μM (MCF-7 cells) or 12 μM (A549 cells) EM-2, and incubation for another 24 h, then proteins were detected by Western blotting. **(E)** Cells were pretreated with 10 μM SP600125 for 6 h, followed by exposure to 8 μM (MCF-7 cells) or 12 μM (A549 cells) EM-2, and incubation for another 24 h, then proteins were detected by Western blotting. **(F)** A549 cells were pretreated with 5 mM NAC for 6 h, followed by exposure to 12 μM EM-2, and incubation for another 24 h, then proteins were detected by Western blotting.

Generally, increased ROS generation causes the activation of ERKs, JNKs, or p38 MAPKs, which further promotes cell apoptosis (Son et al., 2011). To determine whether EM-2-induced ROS production contributed to the activation of MAPK signaling pathway in both A549 and MCF-7 cells, transcriptome profiling by RNA-Seq was conducted. Kyoto Encyclopedia of Genes and Genomes (KEGG) pathway enrichment analysis result showed that MAPK signaling pathway was significantly altered upon treatment with EM-2 (Figure 10A). Furthermore, we identified 20 significantly enriched molecular signaling pathways using KEGG Module Enrichment analysis. To reveal the molecular mechanism involved in the activation of MAPK signaling pathway, we then examined JNK, p38 and ERK1/2 signaling pathways (Figure 10B). In parallel, Western blotting results showed that the levels of JNK, p38, ERK remain unchanged while the level of p-JNK, p-p38, p-ERK were upregulated following EM-2 treatment, suggesting that EM-2 activated MAPK signaling pathway to trigger apoptosis in LC and BC cells (Figure 10C).

To further confirm this result, SB203580 (p38 inhibitor) and SP600125 (JNK inhibitor) were used. Consistent with above results, pretreatment with SB203580 dramatically suppressed the EM-2-induced upregulation of p-p38, p-p53 (S15), p21, and CL-PARP levels (Figure 10D). Moreover, pretreatment with SP600125 attenuated the EM-2-induced upregulation of p-JNK, CL-PARP in both A549 and MCF-7 cells (Figure 10E).

Lastly, we investigated whether ROS induction was the upstream regulator of MAPK pathway or not. Pretreatment with NAC significantly reversed EM-2-induced increase of p-JNK, p-p38, CL-caspase-9, CL-PARP expression and the decrease of PARP and Caspase-9 expression, while it had no effect on the expression level of p38 in A549 cells (Figure 10F). Together, these findings suggested that EM-2 promoted apoptosis via ROS-mediated MAPK signaling pathway.

## 4 Discussion

Herein, EM-2 was identified as the most effective inhibitor against carcinoma cells among the 17 monomers isolated from *Elephantopus mollis* H.B.K. It could suppress the proliferation of A549 and MCF-7 cells, with significant inhibition of colony formation and DNA synthesis due to the induced apoptosis and incomplete autophagy. On one hand, EM-2 inhibited autophagy via repressing autolysosomes acidification, which subsequently induced ER stress-mediated apoptosis in A549 and MCF-7 cells. On the other hand, autophagy inhibition induced by EM-2 was responsible for the intracellular ROS generation. ROS accumulation further triggered G2/M cell cycle arrest via ATM-Chk2-p53-p21 pathway, while it also promoted apoptosis through ROS-MAPK-Bcl-2 pathway in LC and BC cells.

Activation of apoptosis-related signal transduction pathways plays an essential role in cancer therapy (Wang et al., 2019). In the present study, we found that the cleaved forms of Caspase-9 and PARP protein were increased in cells following EM-2 treatment, while Z-VAD-FMK significantly attenuated EM-2-induced increase of CL-PARP and decrease of PARP, revealing that EM-2 triggered apoptosis in A549 and MCF-7 cells. Generally, Bcl-2 family is critical for the regulation of cell apoptosis (Kang and Reynolds, 2009) and contains both anti-apoptosis proteins (including Bcl-2, Mcl-1 and Bcl-xl), and pro-apoptosis proteins (including Bak, Bax, Bim, Bid and PUMA) (Sochalska et al., 2015). Bid, as one of the pro-apoptosis proteins, is the downstream of Caspase-8. After cleaved by Caspase-8, Bid is transferred to mitochondrial membrane, which further induces mitochondrial damage, resulting in the downregulation of mitochondrial membrane potential and eventually the activation of endogenous apoptosis. Consistent with previous observations, we demonstrated that, the expression levels of Bcl-2, Mcl-1 and Bid were downregulated, whereas those of Bax, Bak, Bim, and PUMA were upregulated upon treatment with different concentrations of EM-2, indicating that EM-2 induced apoptosis via the mitochondrial endogenous pathway in LC and BC cells.

Autophagy, a highly conserved lysosomal-dependent degradation process in the evolution of eukaryotes, is characterized by the fusion of lysosomes with autophagosomes, and then degradation of the contents of autophagosomes by the acid enzymes in lysosomes (Levine and Klionsky, 2004). LC3-I, which is generated by proteolytic cleavage of pro-LC-3 by Atg4, can conjugate with phosphatidylethanolamine and subsequently form LC3-II to trigger autophagy (Tan et al., 2019), and the accumulation of LC3-II is often considered as the marker of autophagy. In this study, we found that EM-2 could induce autophagy in A549 and MCF-7 cells with the evidence of increased LC3-II expression. However, the expression of p62 was increased in a dose- and time-dependent manner upon EM-2 treatment, which indicated that, autophagy inhibition induced by EM-2 was a result of selective impairment of autolysosome acidification by p62 (Rubinsztein et al., 2012). Furthermore, acidification of autolysosomes is required for cathepsin activation, the most studied lysosomal proteases that participate in autophagic degradation, which is critical for the maintenance of autophagic flux (Masud Alam et al., 2016). After inhibiting the acidification of autolysosomes, procathepsin D could not be cleaved to form mature cathepsin D upon EM-2 treatment, indicating that EM-2 induced autophagy inhibition via supressing the autolysosome acidification. Alam et al. reports that the impaired autophagy triggers apoptosis via activating ER stress in primary effusion lymphoma both *in vitro* and *in vivo* (Kawaguchi et al., 2011), while another study suggests that combined treatment with bortezomib and bafilomycin A1 (Baf-A1) induces autophagy inhibition and subsequently promotes ER stress-mediated apoptosis (Li et al., 2018). Our findings provided two separate lines of evidence supporting the relationship between autophagy inhibition and ER stress-mediated apoptosis after EM-2 treatment. On one hand, we demonstrated that ER stress was induced in response to the impaired autophagy, as evidenced by an obvious increase in the expression levels of BiP, PDI, and CHOP with increasing concentration of EM-2. On the other hand, GO-BP Enrichment analysis results suggested that EM-2 activated the biological processes of PERK-mediated unfolded protein response and IRE1-mediated unfolded protein response, which led to ER stress induction, and eventually intrinsic apoptotic signaling pathway activation. These results indicated that EM-2 induced impaired autophagy by inhibiting autolysosomes acidification and eventually triggered ER stress-mediated apoptosis, which partially contributed to the LC and BC cell proliferation inhibition.

Recently, accumulating evidences suggest that the function of defective autophagy under metabolic stress is to inhibit the removal of damaged organelles and accumulated proteins, which results in the upregulation of intracellular ROS levels (Schumacker, 2006). As expected, EM-2 blocked autophagy flux, promoted the accumulation of ROS, and subsequently activated cellular oxidative stress response in A549 and MCF-7 cells. Previous studies suggest that ROS generation, induced by anticancer agents, is associated with G2/M cell cycle arrest (Schumacker, 2006), while ATM may be the downstream effector of ROS (Park et al., 2019). Consistent with the previous observations, we found that EM-2 induced G2/M arrest. Mechanistically, Cyclin B1, a key G2/M phase regulator, increased after EM-2 treatment. Cyclin B1 binds to CDK1 (Cdc2) to form the Cyclin B1-CDK1 complex, which becomes activated through coordinated Thr161 phosphorylation coupled with dual Thr14/Tyr15 dephosphorylation, thereby driving G2/M transition. However, in our study, EM-2 treatment increased the expression of both Myt1 and p-Wee1, which leading to elevate p-Cdc2 (Tyr15) levels. And the increased level of p-Cdc2 (Tyr15) prevents the conformational activation of Cdc2’s ATP-binding site, thereby locking Cdc2 in an inactive state. Consequently, this suppresses the activity of the Cyclin B1-CDK1 complex, ultimately leading to G2/M phase arrest. Furthermore, the levels of p-ATM, p-Chk2, p53, p-p53 (S15) and p21 were upregulated in both A549 and MCF-7 cells upon EM-2 treatment, while NAC pretreatment significantly attenuated the upregulation of p-p53 (S15) and p21 in A549 cells. These results revealed that EM-2 induced G2/M phase arrest via ATM-Chk2-p53-p21 pathway in a ROS-dependent manner.

Multiple lines of evidence have shown that, the downstream effectors of ROS may involve different signaling protein, such as JNK and p38, which promotes cell apoptosis (Zhu et al., 2018). Based on the results of KEGG pathway enrichment results, hierarchically clustered heatmap and Western blotting results, we found that EM-2 activated MAPK signaling pathway, with the molecular mechanism focusing on MAPK (JNK) signaling, MAPK (p38) signaling and MAPK (ERK1/2) signaling. Besides, ERK signaling is usually associated with cell proliferation while JNK and p38 signaling are involved in cell death process (Arthur and Ley, 2013). In the present study, we demonstrated that the levels of p-JNK, p-p38, p-ERK were dramatically increased following EM-2 treatment, and pretreatment with SB203580 and SP600125 significantly attenuated the increase of p-p38 and p-JNK expression in both LC and BC cells. Moreover, EM-2-induced increase of p-JNK and p-p38 were significantly inhibited after treatment with NAC in A549 cells. Taken together, these data demonstrated that EM-2 induced MAPK-mediated apoptosis in a ROS-dependent manner.

## 5 Conclusion

**FIGURE 11:**
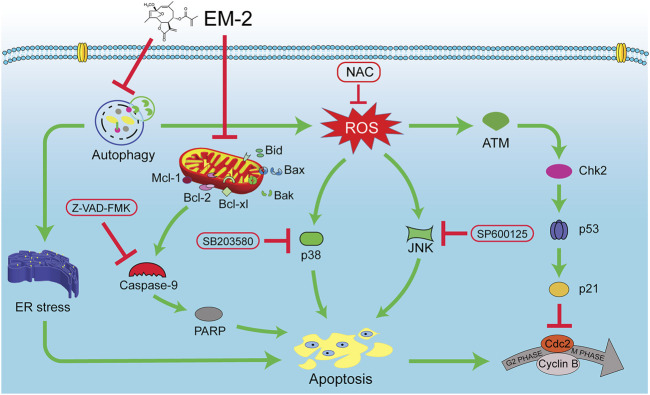
A schematic representation of the molecular mechanism underlying EM-2 inhibiting the proliferation of LC and BC cells. EM-2 inhibited autophagy and then simultaneously triggered ER stress-mediated apoptosis and ROS-mediated apoptosis. Furthermore, the accumulation of ROS enhanced apoptosis via activating ATM-Chk2-p53-p21 pathway and MAPK pathway.

In summary, our findings reveal that EM-2, identified as the most potent anti-cancer compound among the *Elephantopus mollis* H.B.K. monomers, significantly inhibits the proliferation of LC and BC cells by inducing cell apoptosis. Mechanistically, EM-2 induces the impaired autophagy by inhibiting autolysosomes acidification and triggers ER stress-mediated apoptosis in A549 and MCF-7 cells. Additionally, the impaired autophagy induced by EM-2 also promotes ROS production. Furthermore, the accumulation of ROS induces G2/M cell cycle arrest via activating the ATM-Chk2-p53-p21 pathway, and augments apoptosis via activating MAPK-mediated signaling pathway in A549 and MCF-7 cells. Finally, EM-2 also can enhance cell apoptosis via the mitochondrial signaling pathway. These results indicate that EM-2 possesses great potential as a promising drug candidate for the future treatment of LC and BC (Figure 11).

## Data Availability

The datasets presented in the study are deposited in the GEO repository, accession number GSE270062.

## Abbreviations

LC, lung cancer; BC, breast cancer; DMSO, dimethylsulfoxide; MAPK, Mitogen-activated protein kinase; ROS, Reactive Oxygen Species; NAC, N-acetyl cysteine; Baf-A1, Bafilomycin A1; HPLC, high performance liquid chromatography; MTT, 3-(4,5-dimethyl-thiazol-2-yl)-2,5-diphenyltetrazolium bromide; EdU, 5-Ethynyl-2′-deoxyuridine; FITC, fluorescein isothiocyanate; DCFH-DA, Redox-sensitive fluorescent probe 2’-,7′-dichlorofluorescein diacetate; RNA-Seq, RNA Sequencing.
